# A combination of subcuticular suture and enhanced recovery after surgery reduces wound complications in patients undergoing hepatectomy for hepatocellular carcinoma

**DOI:** 10.1038/s41598-018-31287-8

**Published:** 2018-08-28

**Authors:** Zu-Shun Chen, Shao-Liang Zhu, Lu-Nan Qi, Le-Qun Li

**Affiliations:** Department of Hepatobiliary Surgery, Affiliated Tumor Hospital of Guangxi Medical University, Nanning, 530021 China

## Abstract

The aim of this study was to examine whether using subcuticular sutures during initial hepatectomy for hepatocellular carcinoma is associated with shorter postoperative length of hospital stay (PLOS) than using staples for patients treated in the enhanced recovery after surgery (ERAS) approach. A total of 376 patients were randomized to receive either subcuticular sutures or staples (188 per group), and the two groups were compared in terms of the incidence of wound complications and PLOS. Independent risk factors for PLOS were identified by multivariate analysis. Sutures were associated with significantly lower incidence of wound infection (4.3% *vs*. 13.3%, *P* = 0.020) and significantly shorter PLOS (7.97 *vs*. 8.45 days, *P* = 0.048). Independent risk factors for wound infection after hepatectomy were advanced age, increased preoperative body mass index, decreased preoperative serum albumin, and skin closure using staples. These results suggest that subcuticular sutures may be more effective than staples for conducting hepatectomy in patients with hepatocellular carcinoma who receive ERAS care.

## Introduction

In recent decades, the approach of enhanced recovery after surgery (ERAS) has become popular for several abdominal procedures^1,2^, where rapid rehabilitation, prompt discharge, and aesthetic wound healing are important. One of the most frequent causes of longer hospitalization is postoperative wound infection^3^, which adds substantially to healthcare costs and lowers patients’ quality of life by aggravating wound pain and causing cosmetic problems^4^. At least one study has suggested that as many as 30.9% patients undergoing hepatobiliary surgery subsequently suffer wound infection^5^.

Several studies suggest that closing skin incisions with subcuticular sutures rather than staples can reduce incidence of postoperative wound infection and associated complications^3,6,7^. Although staples are fast and easy to use for skin closure following gastrointestinal surgery, subcuticular sutures are associated with better cosmetic outcomes and do not need to be removed later.

We wished to examine whether using such sutures could lead to shorter postoperative length of hospital stay (PLOS) than using staples in patients undergoing hepatectomy to treat hepatocellular carcinoma (HCC) within the ERAS care regime. We also compared sutures and staples in terms of the rate of postoperative wound infection, since such infection is the most frequent cause for prolonged PLOS. We performed multivariate analysis to identify independent risk factors of wound infection after hepatectomy. To the best of our knowledge, ours is the first study to examine the effect of subcuticular sutures on PLOS within the ERAS approach.

## Patients and Methods

### Ethics Statement

This study was conducted in accordance with the Declaration of Helsinki, and the protocol was approved by the Ethics Committee of the Affiliated Tumor Hospital of Guangxi Medical University. Written informed consent was given by all participants for their clinical records to be used in this study.

### Patients and randomization

This prospective study involved 376 patients with HCC who were admitted for initial hepatectomy to the Department of Hepatobiliary Surgery at the Affiliated Tumor Hospital of Guangxi Medical University between January 2012 and July 2017. All these patients fulfilled the following inclusion criteria: (1) they were between 18 and 65 years old, and they were conscious, lucid and able to answer questions; (2) they had been definitively diagnosed with HCC; and (3) they did not have severe organ dysfunction, hypertension, or diabetes. Patients were excluded if they had previously undergone hepatic artery chemoembolization or radiofrequency ablation. They were also excluded if their hepatectomy was laparoscopic.

The 376 patients were randomized into 8 blocks, which were then allocated randomly to receive subcuticular sutures or staples during hepatectomy (188 patients per group). Block randomization allowed us to eliminate effects of differences in admission time and ensure that the two intervention groups would have the same size.

### Hepatectomy and methods for sutures of the incision

The hepatectomy technique was performed as described^8,9^. In the group receiving staples, metallic skin staples were applied 10–15 mm apart. In the group receiving subcuticular sutures, the fascia was closed using interrupted sutures with 0 or 1 absorbable monofilament polydioxanone suture (PDS-II, Ethicon), and the subcutaneous space was irrigated with saline without antibiotics. The subcutaneous fat layer was not sutured, nor was a subcutaneous drain placed. In the subcuticular suture group, surgeons used interrupted subcuticular sutures with a 4-0 monofilament absorbable polydioxanone suture (PDS-II, Ethicon). The suture interval was 15–25 mm, and suture bite length was 15–25 mm from the edge of the skin. This provided tight skin closure.

Average blood loss during surgery was 400 ml or less, and autotransfusion was performed.

### ERAS program

The same ERAS program was applied to both groups of patients (Table 1). ERAS involved a multidisciplinary team of surgeons, nurses, physical therapists, and operating room personnel. Patients were educated preoperatively about ERAS, and they did not need to empty their bowels beforehand. On the day before surgery, they were actively warmed with a coat, their central venous pressure was confirmed to be <5 mmHg, and they received warm fluids. Less intravenous fluid was delivered postoperatively than in conventional care, and intravenous fluid delivery was discontinued earlier. Nasogastric tubes were also removed earlier, and the peritoneal cavity was drained earlier. Early after surgery, patients resumed drinking and oral feeding and were encouraged to become mobile. Patients also received simo decoction and acupuncture at the *tsusanli* acupoint, which we have found to reduce incidence of postoperative ileus and shorten PLOS for HCC patients after hepatectomy^10^. Patients also received continuous portable analgesia epidurally or via a fentanyl transdermal system instead of via a patient-controlled intravenous analgesia pump.

**Table 1:** ERAS program applied to all patients in this study.

**Day before surgery**
Patient education about ERAS
No pre-anesthetic medication
No bowel preparation
**Day of surgery**
CVP monitoring (CVP < 5 mmHg)
Carbohydrate drinks up to 2 h before surgery
Placement of thoracic epidural catheter (T8–T9 level) with continuous infusion of bupivacaine 0.125% with fentanyl l–2 lg/ml at a rate of 4–6 mL/h until day 3, plus intravenous paracetamol or NSAIDs
Nasogastric tube removed within 24 h after surgery
Minimal use of abdominal drain
Active warming with a warmer coat and warmed fluids
Antibiotic prophylaxis 30 min before surgery
**Postoperative day 1**
Continue portable epidural analgesia or analgesia via a fentanyl transdermal system
Mobilization in bed <2 h
Physical therapy four times per day
Reduction of intravenous fluids
Drinking at least 1.0 L liquid
Simo decoction combined with acupuncture at the *tsusanli* acupoint until first flatus
**Postoperative day 2**
Continue portable epidural analgesia or analgesia via fentanyl transdermal system
Try to mobilize out of bed and mobilize in bed <4 h
Reduction of intravenous fluids
Drinking at least 1.5 L of liquid
Remove drainage of peritoneal cavity
Simo decoction combined with acupuncture at the *tsusanli* acupoint until first flatus
**Postoperative day 3**
Epidural catheter removed, NSAIDs started
Mobilization out of bed <6 h
Continuous reduction of intravenous fluids
Semiliquid diet
**Postoperative day 4**
Control pain with oral analgesia only
Mobilization out of bed >6 h
Switch all medications to oral route
Discontinuation of intravenous fluids
Normal diet
**Postoperative day 5**
Encourage full mobilization
Control pain with oral analgesia only
Normal diet
**Postoperative day 6**
Encourage full mobilization
Normal diet
Begin to check discharge criteria
**Postoperative day 7**
Full mobilization
Normal diet
Check discharge criteria
**Postoperative day 8**
Continue as on day 7

*Abbreviations*: CVP, central venous pressure; ERAS, enhanced recovery after surgery; NSAIDs, nonsteroidal anti-inflammatory drugs.

Throughout the hospitalization period before and after surgery, patients were not allowed to smoke, and respiratory inhalation therapy was given before the operation.

### Outcomes assessment

The outcomes were PLOS, wound infection, and wound separation.

We adopted the definition of superficial surgical site infections established by the US Centers for Disease Control^11^ as infections involving only skin or subcutaneous incisional tissues that occur within 30 days of surgery. Such infections must satisfy at least one of the following criteria: (a) purulent drainage from superficial incisions; (b) presence of microorganisms in an aseptically obtained fluid culture or from superficial incisional tissue; or (c) a sign or symptom of infection such as pain, sensitivity, localized swelling, inflammation or heat in superficial incisions intentionally caused by the surgeon (unless the incision was culture-negative).

### Discharge criteri

Functional recovery was defined as adequate pain control requiring only oral analgesia, adequate oral intake with no intravenous fluid requirement, independent mobility sufficient to perform activities of daily life, and blood test values for liver function and inflammatory markers within normal ranges^12^. Patients were assessed daily against these criteria, and an experienced clinician determined readiness for hospital discharge.

### Statistical analysis

Continuous variables were expressed as mean ± standard deviation, and continuous data were compared between the suture and staple groups using Student’s *t* test. Inter-group differences in categorical data were compared using the chi-squared or Fisher exact test. Multinomial logistic regression was used to identify independent risk factors of postoperative wound infection, based on odds ratios (ORs) and associated 95% confidence intervals (CIs). All statistical analyses were performed using SPSS 19.0 (IBM, Armonk, NY, USA). *P* < 0.05 was considered statistically significant.

## Results

### Patient characteristics

Table 2 summarizes clinicopathological characteristics of the suture and staple groups. The groups showed no significant differences on any of the baseline variables examined (all *P* > 0.05).

**Table 2:** Comparison of baseline characteristics between the two patient groups.

Characteristic	Subcuticular sutures	Staples	*P*
Age, years	52.33 ± 11.29	52.19 ± 10.95	0.858
Sex, n (%)			
Male	135 (71.8)	131 (69.7)	0.206
Female	53 (28.2)	57 (30.3)	
Preoperative body mass index, kg/m^2^	25.34 ± 5.25	25.13 ± 5.51	0.358
Preoperative serum albumin, g/l	37.6 ± 7.2	38.2 ± 6.5	0.087
Thickness of subcutaneous fat, cm	2.776 ± 0.738	2.690 ± 0.704	0.678
ASA physical status classification			
1	33 (17.6)	32 (17.0)	0.201
2	141 (75.0)	144 (76.6)	
3	14 (7.4)	12 (6.4)	
Child-Pugh classification, n (%)			0.301
A	173 (92.0)	178 (95.0)	
B	15 (8.0)	10 (5.0)	
Operative time, min	180 ± 85	190 ± 80	0.727
Intraoperative blood transfusion, n (%)	18 (9.6)	16 (8.5)	0.129
Duration of antibiotic prophylaxis, days			0.707
1	15 (8.0)	12 (6.4)	
2	121 (64.4)	125 (66.5)	
3	43 (22.9)	40 (21.3)	
≥4	9 (4.7)	11 (5.8)	

### Outcomes

**Figure 1:**
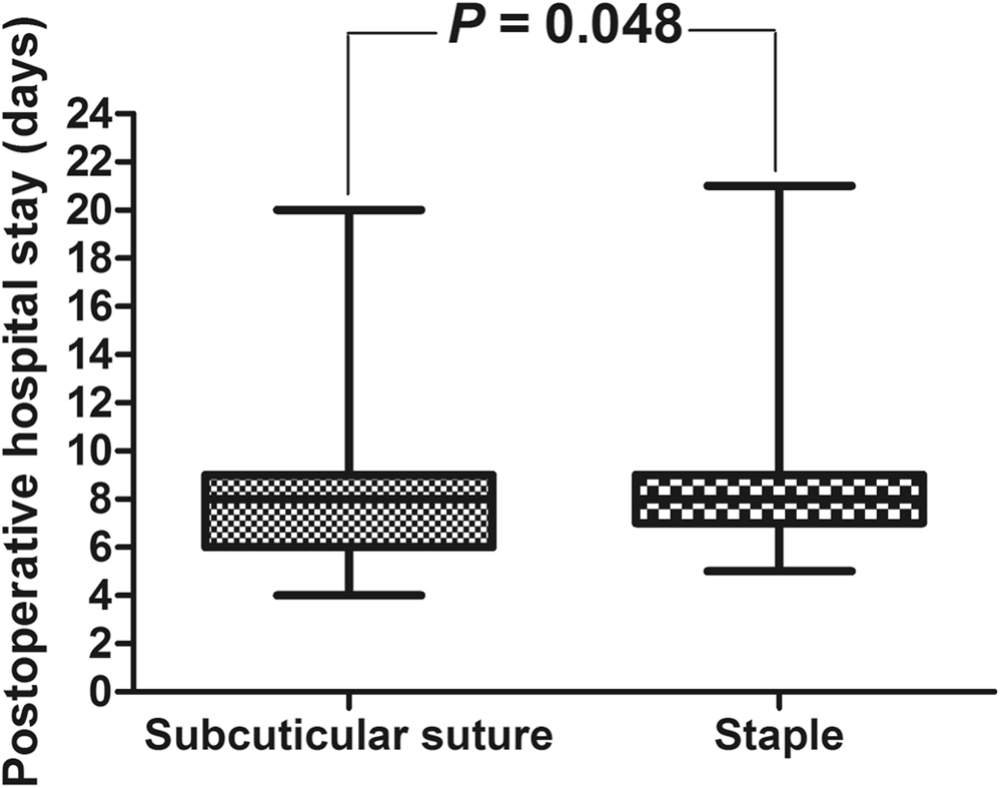
Total length of postoperative hospital stay in the subcuticular suture and staple groups.

The suture group showed significantly better outcomes than the staple group (Table 3), including lower incidence of wound infection (4.3% *vs*. 13.3%, *P* = 0.020) and wound separation (2.7% *vs*. 6.4%, *P* = 0.082). The suture group also showed significantly shorter PLOS (7.97 vs. 8.45 days, *P* = 0.048; Fig. 1).

**Table 3:** Comparison of outcomes between the two patient groups.

Outcome	Subcuticular sutures	Staples	*P*
Wound infection, n (%)	8 (4.3)	25 (13.3)	0.020
Wound separation, n (%)	5 (2.7)	12 (6.4)	0.082
Postoperative hospital stay, days	7.97 ± 2.43	8.45 ± 2.30	0.028

### Risk factors of wound infection after hepatectomy

Multivariate analyses of 11 variables identified the following as independent risk factors of wound infection after hepatectomy (Table 4): advanced age (OR 1.05, 95%CI 1.07–1.14), increased preoperative body mass index (OR 1.15, 95%CI 1.05–1.38), decreased preoperative serum albumin (OR 1.32, 95%CI 1.24–1.56), and skin closure using staples (OR 1.21, 95%CI 1.38–2.27).

**Table 4:** Multivariate analysis to identify risk factors of wound infection after hepatectomy.

Factor	Odds ratio	95% confidence interval	*P*
Advanced age (years)	1.05	1.07–1.14	0.016
Increased preoperative body mass index (kg/m^2^)	1.15	1.05–1.38	0.002
Decreased preoperative serum albumin (g/L)	1.32	1.24–1.56	0.032
Use of staples	1.21	1.38–2.27	0.012

## Discussion

ERAS, which strives to accelerate rehabilitation and discharge, can benefit patients undergoing hepatectomy for HCC, such as by shortening PLOS^13^. However, postoperative wound infection can work against ERAS and prolong PLOS. Here we explore whether using subcuticular sutures instead of staples can reduce the incidence of wound infection after hepatectomy and thereby shorten PLOS. We provide the first evidence that, indeed, using sutures can reduce incidence of postoperative wound infection and concomitantly shorten PLOS as part of the ERAS approach.

Subcuticular sutures are already widely used for skin closure in several types of surgery^6,14–16^. In fact, subcuticular sutures rather than staples are indicated for skin closure in certain types of surgery, such as clean (class 1) surgery^6,7,17^. Subcuticular sutures are particularly popular in plastic surgery because of the relatively low incidence of wound complication and the good aesthetic appearance of the wound^18–20^. Here we demonstrate that subcuticular sutures can be safe and effective for skin closure in hepatobiliary surgery.

The incidence of wound infection in our suture and staple groups is similar to the incidence reported in other studies (5.5–19.2%)^21–25^. We observed sutures to be associated with significantly lower incidence of wound infection than staples. One reason may be that subcuticular sutures allow greater blood flow to cutaneous wounds, which promotes wound healing. One study using infrared laser Doppler flowmetry to calculate blood flow to abdominal incisions in patients after closure with staples, mattress sutures, or subcuticular sutures found postoperative blood flow to be highest in the subcuticular suture group^26^. Those authors suggested that staples and mattress sutures can cause tissue ischemia, perhaps because their closure area affects a greater skin area. In the current study, subcuticular sutures had a bite of 15–25 mm from the skin edge, with the needle introduced at the dermo-epidermal junction^27^. This effectively brings together the dermo-epidermal junction and dermis, both of which contain capillaries, type IV collagen, and fibronectin, all of which are essential for wound healing^28,29^.

We also observed significantly lower incidence of wound separation in the subcuticular suture group than in the staple group. We chose 4/0 PDS II as the material for the subcuticular sutures^30^ because it retains 74% of its tensile strength for up to two weeks and 50% after four weeks. Our results support the idea that this suture is effective for closing wounds requiring prolonged tensile strength following hepatectomy.

Our multivariate analysis identified the following risk factors of wound infection after hepatectomy: advanced age, increased preoperative body mass index, decreased preoperative serum albumin, and skin closure with staples. Several of these factors and others have been linked to wound infection following other types of surgery: advanced age^22^, body mass index^21^, increased operative time^23^, use of silk sutures^23^, increased blood loss^22,31^, postoperative complications, hyperglycemia^21^, and bile leakage^21,23^. Other work has also implicated that perioperative smoking cessation intervention reduces wound infections^32^. Thus smoking may not largely contribute to our results because smoking was banned throughout the hospital stay and respiratory inhalation therapy was given before hepatectomy. Other studies on risk factors of postoperative wound infection have not systematically examined or even reported the method of skin closure, so it is difficult to compare our results with theirs. Future work may wish to examine whether risk factors differ among different patient populations.

The current study contains several limitations that may affect the results and conclusions. First, we did not assess patient satisfaction, patient preference or potential overall effects on the healthcare system, including actual treatment costs. Second, we emphasize that some aspects of the ERAS approach in this study are not widely practiced, such as the use of a nasogastric tube, prophylactic perioperative antibiotics, or post-hepatectomy peritoneal drain. These procedures were standard practice in our hospital at the time of our study. Future work should clarify whether our results are also valid for ERAS approaches that differ from the procedures applied here.

Despite these limitations, the current study provides the first evidence that subcuticular suturing may be more effective than staples for HCC patients undergoing hepatectomy within an ERAS approach.
